# SIRT6 mitigates doxorubicin-induced cardiomyopathy via amelioration of mitochondrial dysfunction: A mechanistic study implicating the activation of the Nrf-2/FUNDC1 signaling axis

**DOI:** 10.7150/ijms.101520

**Published:** 2025-02-28

**Authors:** Qi Wang, Hongshuo Shi, Haowen Zhuang, Guangtong Dong, Kuo Gao, Leilei Liu, Hao Zhou, Yifeng Nie, Junyan Wang, Li Liu

**Affiliations:** 1 First Affiliated Hospital, Heilongjiang University of Chinese Medicine, Harbin 150040, China.; 2 Shuguang Hospital Affiliated to Shanghai University of Traditional Chinese Medicine, Shanghai, China.; 3 School of Pharmaceutical Sciences, Guangzhou University of Chinese Medicine, Guangzhou, Guangdong,510006, China.; 4 Beijing University of Chinese Medicine, Beijing, 100105, China.; 5 University of Rochester Medical Center Rochester, NY, 601 Elmwood Ave, Rochester, NY 14642, United States.; 6 CAS Center for Excellence in Nanoscience, National Center for Nanoscience and Technology, Beijing 100190, P.R China.

**Keywords:** SIRT6, Nrf-2, doxorubicin cardiomyopathy, necroptosis, mitochondrial biogenesis

## Abstract

Doxorubicin-induced myocardial injury, characterized by myocardial hypertrophy and heart failure (HF), represents a primary contributor to end-stage cardiovascular mortality associated with anthracycline drugs. Prior research has elucidated that SIRT6-mediated oxidative processes and mitochondrial metabolic reprogramming are pivotal in sustaining energy metabolism during mitochondrial damage in cardiomyocytes. In the aftermath of doxorubicin-induced myocardial injury, myocardial hypertrophy and fibrosis exacerbate the impairment of cardiac ejection function, resulting in elevated myocardial oxygen consumption. This condition is accompanied by disrupted ATP production, diminished mitochondrial biogenesis, and inadequate synthesis of new mitochondrial DNA, collectively triggering necroptosis and apoptosis pathways. Our preliminary experimental results have confirmed that SIRT6, associated with traditional medicine, exerts cardioprotective effects. Nevertheless, the interaction between SIRT6 and Nrf-2-mediated mitochondrial biogenesis in the context of doxorubicin-induced HF and myocardial hypertrophy remains inadequately understood. The generation of mitochondria is a key mechanism that is involved in DNA repair and cell cycle management.

## Introduction

Doxorubicin (Dox), also referred to as Adriamycin, is extensively utilized in the clinical management of cancer, alongside various other anthracycline chemotherapeutic agents. Nonetheless, Dox therapy is frequently associated with adverse effects, notably multi-organ damage, with cardiotoxicity being the most critical, leading to cardiac injury. The cardiotoxic effects of Dox have been predominantly linked to arrhythmias, ventricular wall dysfunction, and compromised cardiac ejection performance^1^. Research indicates that among cancer patients aged over 65 undergoing anthracycline-based chemotherapy, the incidence of myocardial injury or cardiac ejection dysfunction can reach up to 10%^2^. Although anthracyclines demonstrate significant efficacy in cancer treatment, their pronounced cardiotoxicity is acknowledged as a major contributor to the progression of end-stage cardiovascular disease. Consequently, the investigation of targeted therapeutic interventions for these conditions is of pressing importance.

Mitochondrial metabolic reprogramming constitutes a critical mechanism through which cells modulate their energy production and mitochondrial biogenesis pathways to adapt to diverse environmental stressors^3^. In this context, cardiomyocytes modify the regulatory patterns of their mitochondrial networks to accommodate fluctuations in nutrient availability, oxygen concentration, and proliferative requirements^4^, ^5^. Importantly, this reprogramming can be modulated by changes in oxidative phosphorylation, which facilitates the activation of mitochondrial pathways involved in the synthesis of nucleotides and fatty acids. These mitochondrial metabolic processes not only provide energy to the cell but also contribute to mitochondrial DNA synthesis, thereby influencing cardiomyocyte growth, differentiation, and apoptosis^6^, ^7^.

Current research evidence indicates that the histone deacetylase SIRT6 mimics the effects of caloric restriction in promoting lifespan extension and improving glucose homeostasis^8^. As a key regulatory target in cellular energy metabolism, SIRT6 catalyzes the deacetylation of histone H3 to regulate various mitochondrial biological processes, including DNA repair, glucose/lipid metabolism, and inflammation. Previous studies have preliminarily demonstrated that SIRT6 mitigates renal injury by deacetylating and suppressing the expression of ERK1/2^9^-^11^.

Furthermore, reduced levels of SIRT6 have been found in the plasma of cancer patients undergoing anthracycline-based chemotherapy^12^. Clinically, SIRT6 in the bloodstream are currently being investigated as a potential biomarker for assessing health or disease states. Similarly, reduced serum SIRT6 levels have been detected in individuals with stable angina and acute coronary syndrome^1^. Further research examining the clinical relevance of SIRT6 in doxorubicin-induced cardiotoxicity has demonstrated a significant decrease in SIRT6 levels in patients' plasma following four cycles of anthracycline chemotherapy, compared to pre-treatment levels^13^, ^14^. This reduction in SIRT6 expression was correlated with increased plasma lactate concentrations post-treatment, suggesting enhanced glycolysis and cellular metabolic activity associated with chemotherapy. These findings imply a connection between reduced SIRT6 expression and increased glycolytic activity with anthracycline-induced toxicity in humans^15^, ^16^. Nonetheless, the regulatory mechanisms governing SIRT6 in mitochondrial metabolic reprogramming and mitochondrial biogenesis dysfunction remain inadequately understood.

Prior research has demonstrated that Sirtuins, a highly conserved class of deacetylases present from bacteria to humans, consist of seven recognized members within the human Sirtuin family: SIRT1 through SIRT7^17^. Furthermore, natural medicines containing ginsenoside components have been shown to stabilize the cytoskeletal microtubules of cardiomyocytes under hypoxic stress via SIRT5 regulation^18^. These compounds also influence FUNDC1-mediated mitophagy and normalize the mitochondrial unfolded protein response, highlighting the therapeutic potential of Sirtuins in addressing mitochondrial dysfunction.

We investigated the mechanisms by which quercetin aids in maintaining mitochondrial quality control in heart cells under the stress of ischemia-reperfusion^19^. In our *in vivo* studies, we employed enzyme-linked immunosorbent assays (ELISA) to assess myocardial injury biomarkers, quantified the transcriptional levels of SIRT5, DNA-PKcs, and MLKL at various time points following reperfusion. *In vitro*, we developed DNA-PKcs gene modification models through adenoviral transfection and isolated primary cardiomyocytes from SIRT5-modified mouse models. We employed various methods to clarify how quercetin regulates the MQC network in cardiomyocytes after I/R injury^19^.

Our findings indicate that I/R injury leads to structural modifications in cardiomyocytes. Quercetin demonstrated cardioprotective properties, with its administration modulating the transcription levels of DNA-PKcs, SIRT5, and MLKL during the progression of I/R injury. *In vitro* studies revealed that quercetin alleviated mitochondrial oxidative stress-induced damage in cardiomyocytes via DNA-PKcs and sustained optimal mitochondrial energy metabolism by modulating autophagy and mitochondrial dynamics. Mechanistically, quercetin stabilized DNA-PKcs through SIRT5-mediated desuccinylation, and this interaction regulated the "autophagy-unfolded protein response," thereby preserving mitochondrial membrane and genome integrity, as well as mitochondrial dynamics and energy metabolism^19^.

Subsequent investigations have demonstrated that I/R injury is correlated with decreased expression of SIRT5, which in turn triggers necroptosis in cardiomyocytes through mitochondrial pathways. Furthermore, there was a marked reduction in mitochondrial membrane potential and FUNDC1-mediated mitophagy levels. Quercetin, a naturally occurring compound, conferred protection to myocardial tissues during I/R injury by enhancing SIRT5 expression. This upregulation mitigated oxidative stress-induced damage to cardiomyocytes and preserved mitochondrial energy metabolism by modulating mitophagy and mitochondrial dynamics.

Furthermore, our study demonstrates that SIRT1 plays a protective role in sinoatrial node cells by preserving mitochondrial morphology and structural integrity, thereby inhibiting the activation of stress-induced pathways. Under conditions of hypoxic stress, SIRT1 facilitates the translocation of Drp1 is directed to the mitochondria, which helps prevent excessive mitochondrial division, triggers mitophagy, and reinstates mitochondrial membrane potential. These protective mechanisms are predominantly mediated through β-tubulin activity^20^. Similarly, quercetin mitigates excessive mitochondrial fission via SIRT1, maintains mitochondrial energy metabolism, and shields sinoatrial node cells from stress-induced damage. Our findings underscore the importance of preserving SIRT1 and cytoskeletal protein activity as a critical strategy for safeguarding mitochondrial function, thereby mitigating sinoatrial node cell injury and offering a potential therapeutic approach for the treatment of arrhythmias.

While the involvement of the SIRT protein family in myocardial and vascular injury has been extensively validated through various pathways, the precise mechanisms and downstream targets in doxorubicin-induced myocardial injury remain unverified. To explore this hypothesis, we conducted both *in vivo* and *in vitro* studies to further elucidate the cardioprotective effects of SIRT6, specifically targeting mitochondrial pathways. These studies also evaluated the influence of SIRT6 on cardiac function and cardiomyocyte functionality in both *in vitro* and *in vivo* models. Additionally, we investigated the molecular mechanisms governing the interaction between SIRT6 and Nrf-2-mediated mitochondrial biogenesis, particularly in relation to the varied responses to doxorubicin-induced cardiotoxicity in cardiomyocytes. Our results elucidate potential therapeutic targets and lay the groundwork for the development of innovative cardioprotective drugs aimed at mitigating doxorubicin-induced myocardial injury.

## Methods

### Raw data processing

The analysis used GEO datasets GSE106297 and GSE157282. In this study, GSE106297 was used as the training set^21^, while GSE157282 was used as the validation set^22^. GSE106297 contains 8 myocardial cell samples with DOX intervention and 4 normal myocardial cell samples. GSE157282 contains 3 myocardial cell samples with DOX intervention and 3 normal myocardial cell samples. Data on mitophagy-related genes comes from the Genecard database, with all GEO dataset information listed in Table 1.

### Data filtering and processing

Accurate mRNA data were derived using Perl-based matching and sorting techniques on the transcriptomic data, and then normalization was applied to GSE106297 and GSE157282. The R package "limma" was used to process and normalize the raw gene expression matrices of the GEO datasets. The SVA package was employed to correct for batch effects between GSE106297 and GSE157282.

### Clustering analysis of DOX-induced myocardial injury samples

By evaluating criteria like the cumulative distribution function curve, consensus clustering score, and consistency matrix, we determined the optimal number of clusters. In this study, we identified k=5 as the maximum number of clusters.

### Co-expression Gene Identification

Using the WGCNA algorithm, genes were classified, and the associations between modules and traits were determined. Co-expression networks were constructed using the top 25% most variable genes from the dataset GSE106297. A dynamic tree cut method with a 0.25 threshold was used to merge modules, after which the most strongly correlated modules from both classification methods were mapped. KEGG^23^ and GO^24^ enrichment analyses were conducted with the clusterProfiler package in R. Additionally, we conducted GSEA to identify potential pathways.

### Animal experiments

All procedures and animal experiments were rigorously conducted in compliance with the Guide for the Care and Use of Laboratory Animals as established by the National Institutes of Health. These procedures received additional approval and authorization from the Experimental Animal Center at Guangzhou University of Chinese Medicine. All animal procedures were approved by the Experimental Animal Ethics Committee of Guangzhou University of Chinese Medicine Laboratory Animal Center (No. GZTCMF-20230097).C57BL/6J mice were procured from the same center. The mice were kept on a 12-hour light/dark schedule with unlimited access to food and water. During the data analysis phase, investigators were blinded to the genotypes of the mice to ensure unbiased results^19^, ^25^.

Subsequent experimental investigations were performed utilizing SIRT6 transgenic (SIRT6^TG^) mice. Both C57BL/6J and SIRT6^TG^ mice were housed under standard conditions. Following a two-week acclimatization period, the mice designated for the DOX-induced myocardial injury model were administered doxorubicin (DOX, D1515, Sigma-Aldrich) at a dosage of 12.5 mg/kg, dissolved in saline, through a single intraperitoneal injection over the course of one week. Mice in the control group received saline injections to serve as a baseline for comparative analysis^26^.

### Cell culture

Our research group isolated and cultured neonatal mouse cardiomyocytes using previously established experimental protocols and procedures. Hearts from sterile neonatal C57BL/6J mice, aged one day, were rapidly excised and subjected to enzymatic digestion using Type I collagenase (LS00419, Worthington, Lakewood, NJ, USA) for several minutes. To eliminate fibroblasts, a differential pre-culture was conducted for 90 minutes. Subsequently, the neonatal cardiomyocytes were re-seeded onto gelatin-coated culture dishes and incubated in a humidified incubator (Thermo Fisher Scientific, Waltham, MA, USA) maintained at 95% air and 5% CO₂ for 48 hours. The culture medium was made up of Dulbecco's Modified Eagle Medium (DMEM, Gibco, Grand Island, NY, USA) with an addition of 10% fetal bovine serum (FBS, Gibco) and 1% penicillin/streptomycin mixture (Gibco). To suppress fibroblast proliferation, 5-bromo-2-deoxyuridine (BrdU) was incorporated into the culture medium. For the pre-treatment with DOX, primary mouse cardiomyocytes and HL-1 mouse cardiomyocytes were co-cultured for a duration of 24 hours in a medium containing doxorubicin dissolved in dimethyl sulfoxide (DMSO) at a predetermined concentration^26^.

### Immunofluorescence detection of mouse myocardial tissue

Fresh cardiac tissue samples were promptly preserved at temperatures of -20°C or -80°C using Optimal Cutting Temperature (OCT) compound. Subsequently, sections with a thickness of 4 µm were prepared from the cryopreserved tissue samples. Frozen sections were fixed at ambient temperature with a 10% formalin solution for 15 minutes for immunofluorescence staining, and then washed three times with phosphate-buffered saline containing Tween 20. To achieve permeabilization, the sections were treated with a 0.5% Triton X-100 solution in PBS. To prevent nonspecific binding, the sections were incubated with 10% goat serum at room temperature for 1 hour.

The tissue sections were treated with primary antibodies and incubated overnight at 4°C. After three more wash steps, they were stained with fluorescence-labeled secondary antibodies. Nuclei were counterstained with DAPI. Sections were imaged with an LSM 510 confocal microscope, and image analysis software was used to quantify the percentage of areas with positive staining for the target antibody. For each experimental group, 6 to 8 fields of heart tissue were analyzed^19^, ^25^.

### Echocardiography

To comprehensively investigate the impact of DOX on cardiac ejection function in murine models, echocardiographic assessments were conducted on various cohorts of wild-type and SIRT6-modified DOX model mice. Precise body weight measurements were obtained for each mouse prior to the induction of anesthesia with 3% isoflurane. To ensure thermal stability, the mice were positioned on a heating pad set to maintain a constant body temperature of 37°C. The thoracic fur was meticulously removed, and ultrasound gel, pre-warmed to body temperature, was applied to the exposed area.

During the procedure, echocardiographic images were obtained by maintaining gentle contact between the echocardiography probe and the chest. Anesthesia was sustained with 1.5% isoflurane throughout the examination to minimize stress and ensure consistent imaging quality^27^.

### RT-PCR testing

Cardiac tissues from each experimental group were used to isolate total RNA, comprising wild-type and SIRT6-transgenic mice subjected to DOX-induced myocardial injury, utilizing the TRIzol reagent in conjunction with the RNeasy Total RNA Isolation Kit. Subsequently, the isolated RNA was reverse-transcribed into complementary DNA (cDNA) employing the iScript cDNA Synthesis Kit. Bio-Rad Laboratories' iQ SYBR Green Supermix was employed for conducting quantitative real-time PCR on the CFX96 Touch Real-Time PCR Detection System^28^.

### Cardiomyocyte immunofluorescence detection

For the immunofluorescence staining of cardiomyocytes, mouse cardiomyocytes were fixed at room temperature using 4% paraformaldehyde for a duration of 15 minutes. Subsequently, the cells underwent permeabilization in PBS containing 0.2% Triton X-100 and 5% fetal bovine serum (FBS) for 1 hour. Post-permeabilization, the cells were incubated overnight at 4°C with the primary antibody. Following thorough washing with PBS, the cardiomyocytes were treated with Alexa Fluor-labeled secondary antibodies. For imaging purposes, the immunofluorescent expression levels of target proteins, such as TOM20 and NLRP3, were visualized using a Leica laser confocal microscope at designated excitation wavelengths. Semi-quantitative analysis of the immunofluorescence signals was conducted utilizing ImageJ software, and the data obtained were analyzed statistically with GraphPad Prism version 9.0.

### ELISA test

Enzyme-linked immunosorbent assay kits, procured from Nanjing Jiancheng Bioengineering Institute, were utilized in accordance with the manufacturer's protocols to quantify inflammatory mediators in cardiomyocytes across various experimental cohorts. Specifically, the following ELISA kits were employed: H002-1-2 for the detection of IL-1β, H009-1-2 for IL-10, and H015-1-2 for IL-18. Furthermore, the assessment of oxidative stress and antioxidant enzyme activities in cardiomyocytes was conducted using the following kits: A003-1-2 for the quantification of malondialdehyde (MDA), 007-1-1 for catalase (CAT) activity, and A001-3-2 for total superoxide dismutase (T-SOD) activity, the latter being measured via the WST-1 method.

### Statistical analysis

Results were expressed as the mean ± standard deviation (SD). Statistical analyses were conducted utilizing Statistical Product and Service Solutions (SPSS, version 1.0) and GraphPad Prism version 9.0 for data processing and quantitative analysis. When data followed a normal distribution, a t-test was used for comparing two groups, and one-way ANOVA was used for comparing multiple groups. Post-hoc analyses were executed using the Bonferroni test or Tamhane's T2 test, contingent upon the satisfaction of the homogeneity of variance assumption. Statistical significance was determined by a P-value under 0.05.

## Results

### Database analysis of SIRT6-mediated doxorubicin-induced myocardial injury phenotypic differences and related mechanisms of action

**Figure 1:**
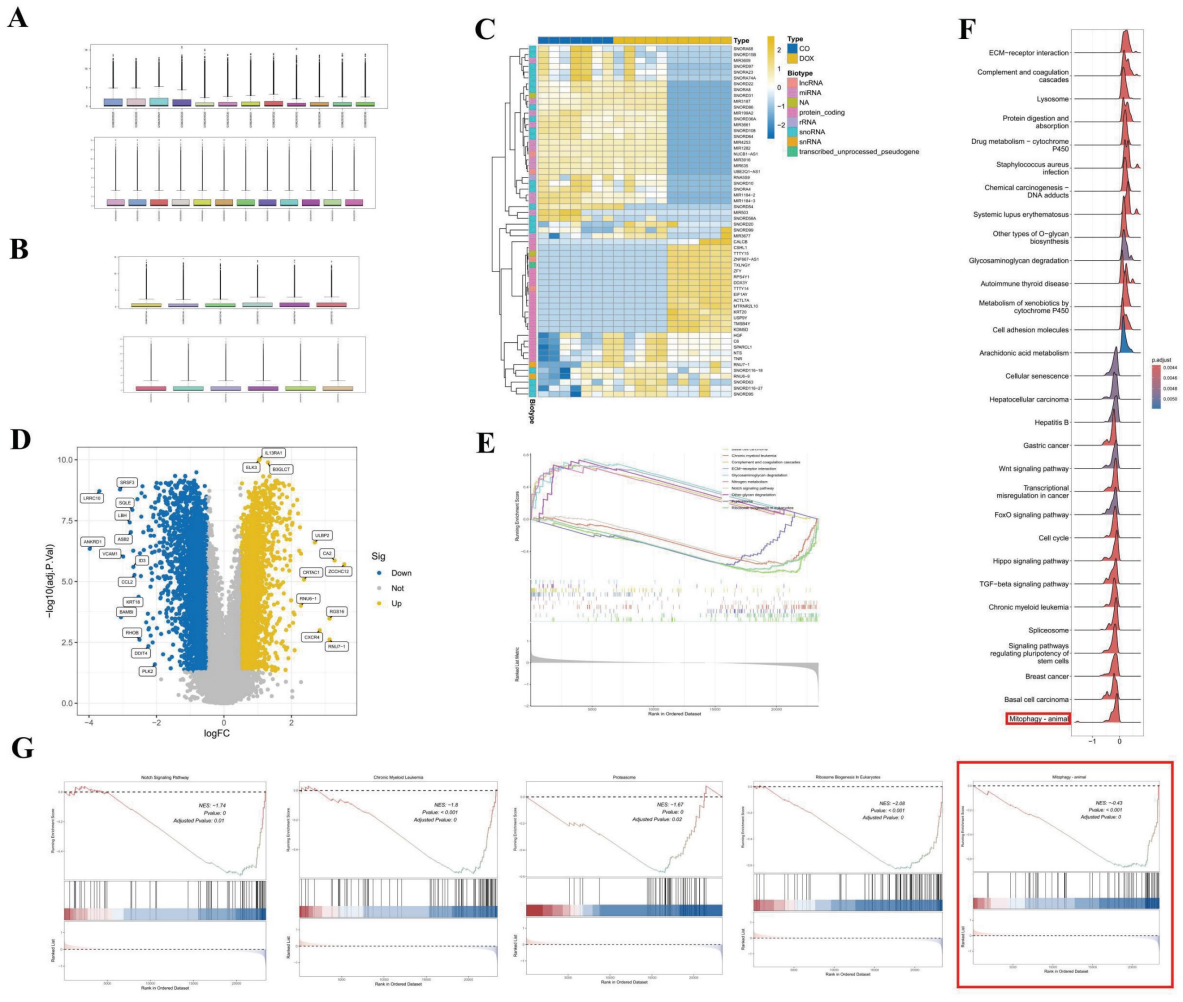
** Data processing and analysis of GSE106297 and GSE157282.** (A) Normalization of GSE106297; (B) Normalization of GSE157282; (C) Heatmap of GSE106297; (D) Volcano plot of GSE106297. (E-G) GSEA analysis.

The normalization procedures applied to datasets GSE106297 and GSE157282 are depicted in Figures 1A and 1B, respectively. Figure 1C illustrates the clustering diagram from the differential analysis of the GSE106297 dataset, which identifies a total of 3,787 differential genes, as detailed in Figure 1D. The outcomes of the GSEA are presented in Figures 1E, F, and G, suggesting that mitophagy may serve as a crucial pathway in DOX-induced cardiomyopathy. Furthermore, we employed WGCNA to identify key gene modules that are closely associated with mitochondrial-related gene (MRG) clusters. Setting the soft-thresholding power parameter β at 12 resulted in an R² value of 0.9, we constructed a scale-free network under these parameters, as shown in Figure S1F. Six modules were identified as significant. The heatmap in Figure S1G displays the Topological Overlap Matrix (TOM) of all genes associated with these modules. The examination of the relationship between the modules and clinical traits (Cluster 1 and Cluster 2) highlighted the importance of the blue module, as shown in Figures S1H, I, and J.

**Figure 2:**
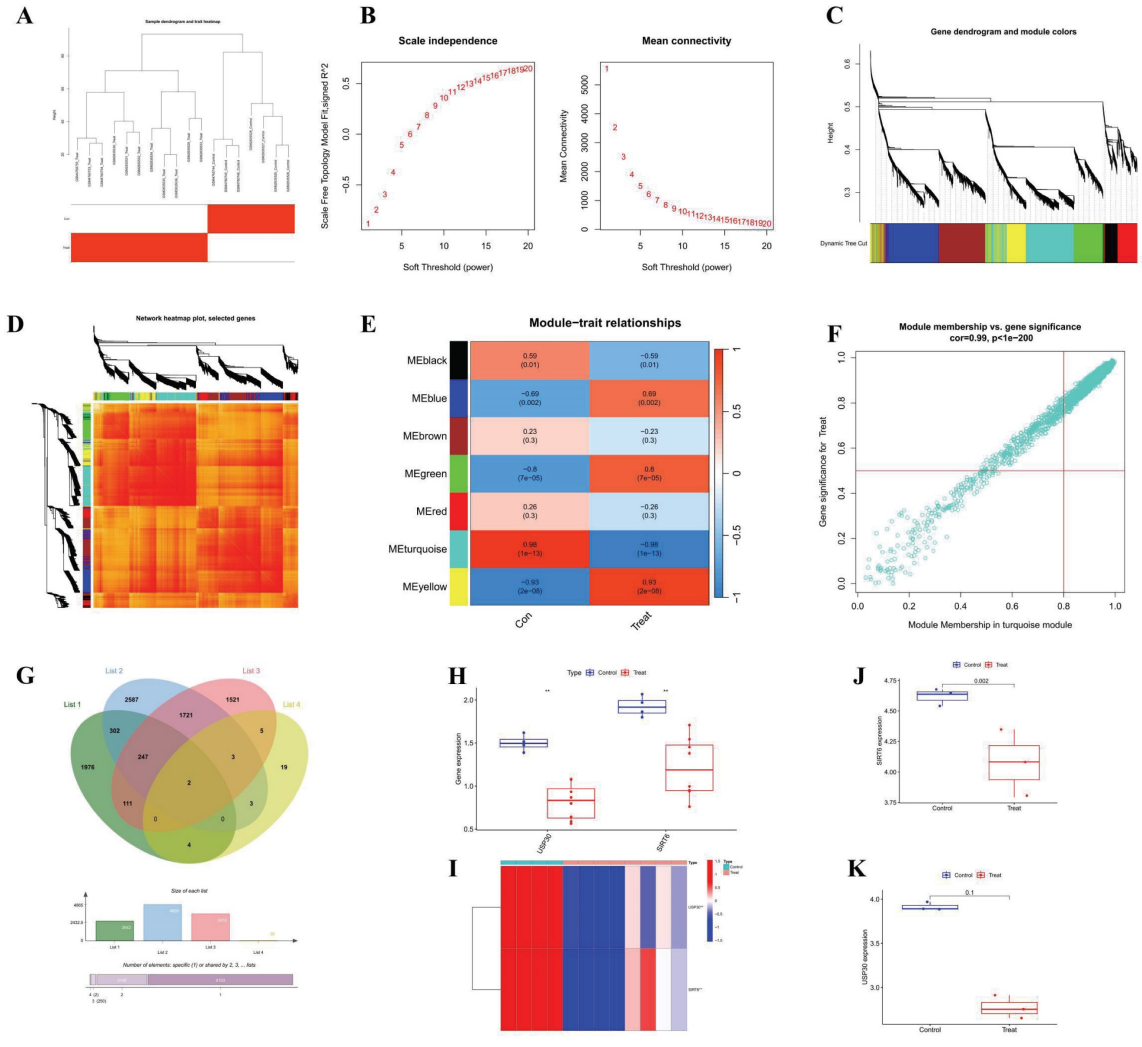
** WGCNA analysis (A) Cluster diagram of module eigengenes.** (B) Set soft threshold power (C-D) TOM heatmap of 6 modules (E) Heatmap of correlation analysis of module eigengenes with clinical features. Rows and columns represent modules and clinical features, respectively (F) Scatter plot of the genetic significance of the blue module members (G) Venn Diagram (H) Differential analysis of GSE106297 (I) Heatmap of GSE106297 (J) Differential analysis of GSE157282 (SIRT6) (K) Differential analysis of GSE157282 (USP30).

We constructed a co-expression network utilizing WGCNA for both the normal control group and the DOX-induced cardiomyopathy samples, leading to the identification of key gene modules. Under these conditions, we discerned the co-expression gene modules, as depicted in Figure 2A. Subsequently, the application of the dynamic tree cutting algorithm resulted in the identification of six distinct co-expression modules, each distinguished by a unique color, and facilitated the generation of the TOM heatmap (Figures 2B-2E). Furthermore, by employing the genes from these six modules, we examined the co-expression similarities and continuity in relation to the clinical characteristics of the modules (normal control and DOX). Our analysis revealed that the association between the emerald module and DOX was the most pronounced (Figure 2F).

**Figure 3:**
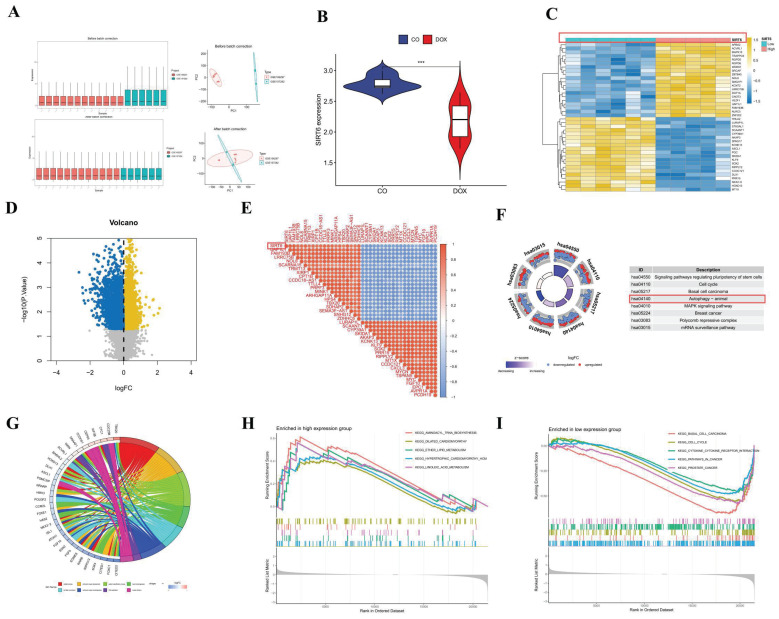
** SIRT6 in gene analysis.** (A) PCA analysis after batch effect removal; (B) The differential expression of gene SIRT6; (C) Differential analysis heatmap; (D) Volcano plot; (E) Correlation analysis heatmap (F) Differential gene KEGG analysis; (G) Differential gene GO analysis; (H) GSEA analysis of downregulated genes; (I) GSEA analysis of upregulated genes.

Through the application of key modules from WGCNA, we identified two genes, SIRT6 and USP30, as depicted in Figure 2G. The expression levels of SIRT6 and USP30 were assessed in the dataset GSE106297 (Figures 3H and 3I) and further validated in the dataset GSE157282 (Figures 2J and 2K). We subsequently integrated two DOX datasets, GSE106297 and GSE157282, to eliminate batch effects and conducted Principal Component Analysis (PCA), as illustrated in Figure 3A. The expression levels of SIRT6 in the integrated dataset are shown in Figure 3B. Utilizing the high and low expression levels of SIRT6, we carried out differential gene expression analysis within the integrated dataset. The outcomes of this analysis are presented in a heatmap (Figure 3C), a volcano plot (Figure 3D), and a correlation heatmap (Figure 3E). Enrichment analysis revealed that the biological processes associated with the differentially expressed genes predominantly involve autophagy (Figure 3F) and oxidative stress (Figure 3G). The high and low expression levels of SIRT6, along with the results from GSEA, are displayed in Figures 3H and 3I.

### The regulatory mechanism of SIRT6 in doxorubicin-induced myocardial injury

**Figure 4:**
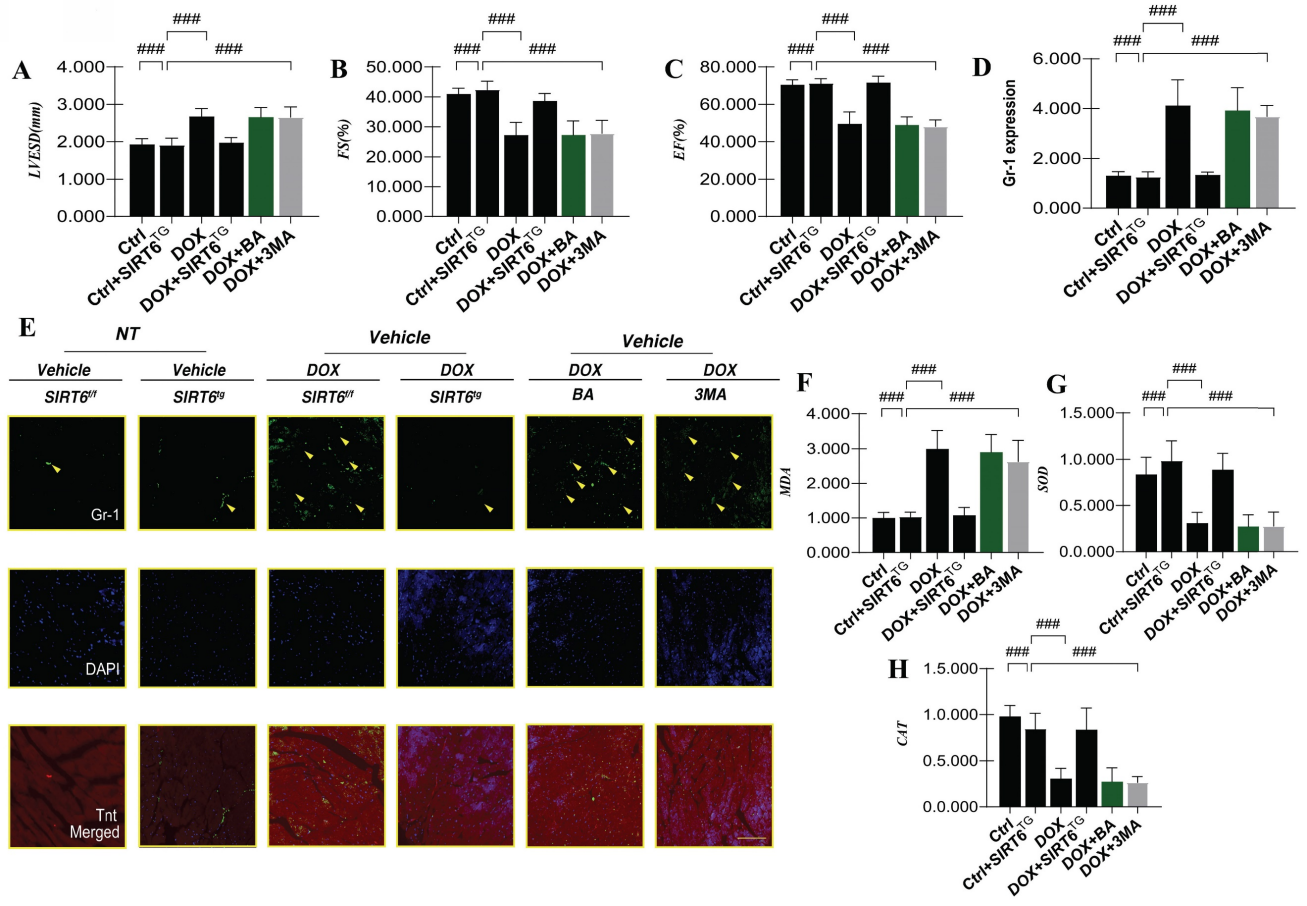
** SIRT6 involvement in DOX-induced myocardial oxidative stress and inflammatory injury.** (A) LVESD: Left ventricular end-systolic diameter (LVESD); (B) FS: Left ventricular short-axis fractional shortening; (C) EF: Left ventricular ejection fraction; (D) Gr-1 expression level; (E) Gr-1 and Tnt dual-fluorescence staining in myocardial tissue; (F) MDA: Expression level of the oxidative stress marker malondialdehyde (MDA); (G) SOD: Expression level of the antioxidant enzyme superoxide dismutase (SOD); (H) CAT: Expression level of the antioxidant enzyme catalase (CAT). Values are presented as mean ± SD. All experiments were performed at least three times. #p < 0.05.

To further elucidate the role and underlying mechanisms of SIRT6 in DOX-induced myocardial injury, we produced a transgenic mouse model featuring SIRT6 and subsequently created a DOX-induced cardiomyopathy model. Our experimental findings demonstrated that DOX-induced myocardial injury resulted in a significant decrease in cardiac ejection fraction (EF) and fractional shortening (FS), indicative of impaired left ventricular systolic function. Furthermore, an increase in left ventricular end-systolic diameter (LVESD) was observed (Figure 4A-C), suggesting that DOX-mediated cardiac dysfunction is associated with the pathological mechanism of myocardial hypertrophy. In conjunction with the decline in cardiac ejection function, there was a marked exacerbation of neutrophil-mediated inflammatory damage within the myocardium (Figure 4D-E).

The analysis of serum samples revealed a marked surge in MDA levels subsequent to DOX-induced myocardial injury, which was accompanied by a pronounced reduction in the activities of the antioxidant enzymes superoxide dismutase (SOD) and catalase (CAT) (Figure 4F-H). Importantly, intervention with SIRT6 transgenics effectively reversed these alterations, leading to an improvement in cardiac ejection function and a reduction in myocardial inflammatory damage following DOX-induced injury. Additionally, SIRT6 was observed to inhibit the expression of mitochondrial oxidative stress markers while enhancing the activity of antioxidant enzymes (Figure 4A-H). These observations point to the essential regulatory influence of SIRT6 in the setting of myocardial injury and cardiac dysfunction induced by DOX.

Subsequent experimental investigations have revealed that interventions utilizing mitochondrial ATP synthase inhibitors and mitophagy inhibitors were ineffective in significantly alleviating DOX-induced damage (Figure 4A-H). This observation implies that mitochondrial oxidative stress damage might manifest during the initial phases of DOX-mediated inflammatory myocardial injury. Nevertheless, the possibility of modulating this pathway through mitophagy and its consequent effects on mitochondrial energy metabolism have yet to be conclusively determined.

### SIRT6 regulates the NLRP3 inflammasome and mediates the alleviation of myocardial inflammatory injury

**Figure 5:**
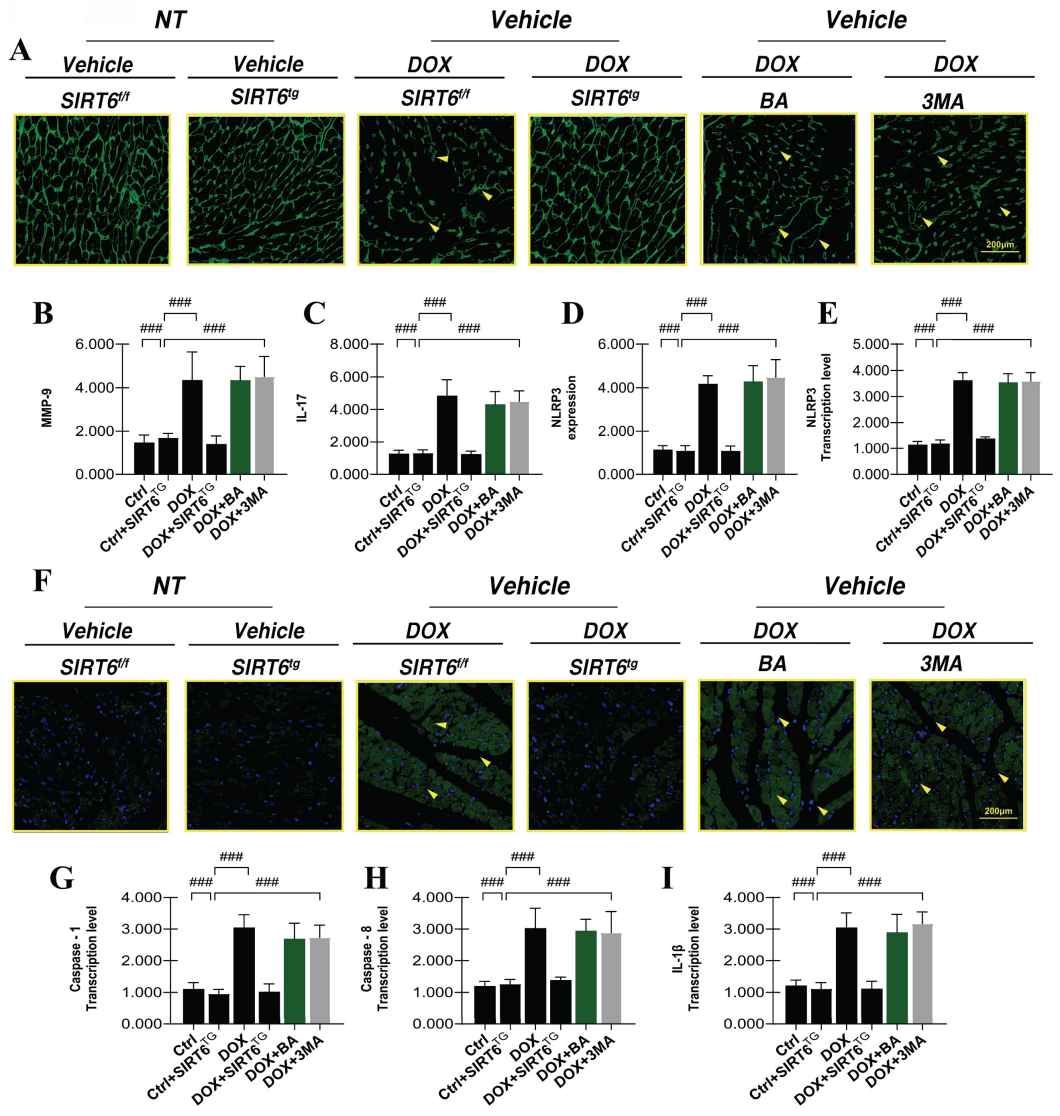
** SIRT6 involvement in DOX-induced inflammatory injury and pyroptosis.** (A) WGA: Detection of myocardial cell hypertrophy; (B) MMP-9: Detection of inflammatory factor expression levels; (C) IL-17: Expression level of the inflammatory cytokine interleukin-17 (IL-17); (D) NLRP3: Expression level of NLRP3 inflammasome; (E) NLRP3: Transcriptional level of NLRP3; (F) NLRP3-immunofluorescence analysis; (G) Caspase-1: Transcriptional level of Caspase-1; (H) Caspase-8: Transcriptional level of Caspase-8; (I) IL-1β: Transcriptional level of interleukin-1 beta (IL-1β). Values are presented as mean ± SD. All experiments were performed at least three times. #p < 0.05.

Research has demonstrated that the cardiotoxic effects of DOX significantly activate and upregulate inflammatory reprogramming during myocardial injury, which in turn leads to pronounced myocardial remodeling and alterations in cardiac ejection function. Under conditions of dual stress, the adaptive and regulatory mechanisms governing myocardial stress responses become dysregulated, ultimately resulting in diminished myocardial activity and cardiac dysfunction. Subsequent experiments have confirmed that DOX-induced myocardial injury is characterized by marked cardiomyocyte hypertrophy and elevated expression levels of the inflammatory factors MMP-9 and IL-17(Figure 5A-C). These results align with earlier research, which have shown that DOX-mediated myocardial injury is associated with increased cardiomyocyte size and structural disruption, both of which are critical factors contributing to myocardial hypertrophy and impaired cardiac ejection function.

In addition to hypertrophy and myocardial injury, the excessive secretion of inflammatory factors can disrupt intracellular homeostasis and impair energy metabolism in cardiomyocytes. Notably, mice in the SIRT6 transgenic group did not exhibit significant cardiomyocyte hypertrophy following DOX-induced damage, the expression levels of MMP-9 and IL-17 were notably decreased (Figure 5A-C). These findings suggest that SIRT6 functions as a critical upstream regulator in mitigating DOX-induced myocardial hypertrophy and inflammatory injury. However, interventions using mitochondrial autophagy inhibitors and mitochondrial ATP synthase inhibitors did not modify the hypertrophic and inflammatory damage phenotypes (Figure 5A-C).

To elucidate the mechanism underlying SIRT6-mediated regulation of myocardial injury and cardiomyocyte programmed cell death, we conducted myocardial immunofluorescence staining for NLRP3 and quantified the transcriptional levels of Caspase-1, Caspase-3, and IL-1β (Figure 5D-I). This was done to evaluate whether DOX-induced myocardial injury activates pyroptosis pathways. A substantial upsurge in fluorescence intensity and NLRP3 transcription levels was observed after DOX treatment. Additionally, there was a marked elevation in the transcription levels of Caspase-1, Caspase-3, and IL-1β. The results indicate that myocardial injury caused by DOX activates the NLRP3 inflammasome, leading to increased pyroptosis, damage to cardiomyocytes, and mitochondrial dysfunction (Figure 5A-I). Notably, interventions using mitochondrial autophagy inhibitors and mitochondrial ATP synthase inhibitors did not modify the NLRP3 inflammasome-mediated pyroptosis phenotype (Figure 5A-I).

### SIRT6 and Nrf-2 regulate mitophagy to alleviate doxorubicin-induced myocardial injury

Previous research has demonstrated that mitophagy, a pivotal mechanism for initiating mitochondrial quality control, is activated in cardiomyocytes in response to DOX-induced cardiotoxicity. Under conditions characterized by excessive accumulation of ROS, substrate deficiency, cellular aging, and damage, there is an accumulation of dysfunctional or damaged mitochondria within cardiomyocytes. Receptor-dependent mitophagy, mediated by FUNDC1, and ubiquitination-dependent pathways, mediated by PINK/Parkin, are instrumental in the removal of these damaged mitochondria. This process is essential for maintaining mitochondrial homeostasis, normalizing the unfolded protein response, and stabilizing both the mitochondrial network and the intracellular environment^29^, ^30^.

**Figure 6:**
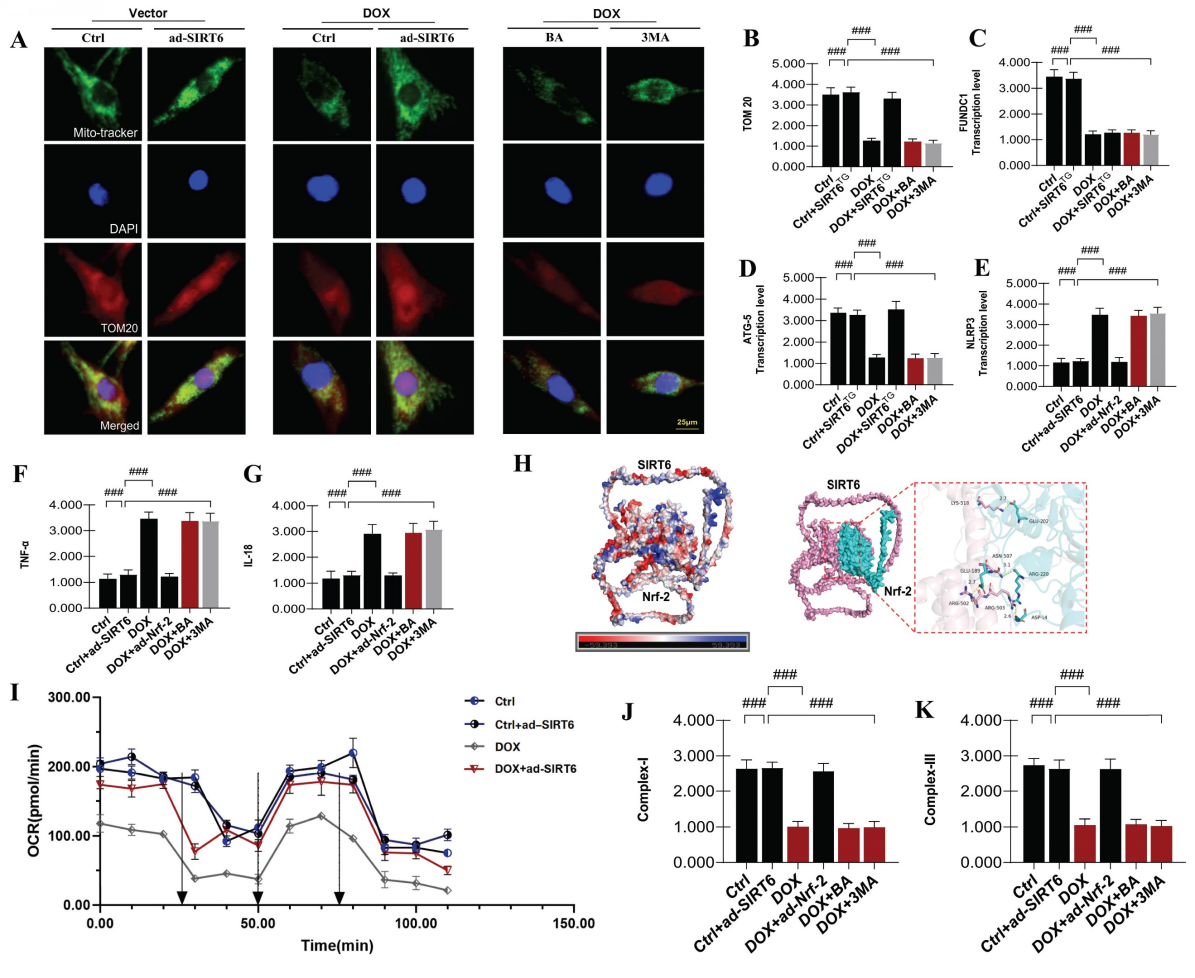
** SIRT6 and Nrf-2 involvement in DOX-induced mitochondrial autophagy and mitochondrial energy metabolism dysfunction.** (A-B) TOM20: Detection of myocardial cell mitophagy levels; (C) FUNDC1: Transcriptional level of FUNDC1; (D) ATG5: Transcriptional level of ATG5; (E) NLRP3: Expression level of NLRP3; (F) TNF-α: Expression level of tumor necrosis factor-alpha (TNF-α); (G) IL-18: Expression level of interleukin-18 (IL-18); (H) Molecular docking experiment verification (SIRT6 and Nrf-2); (I) Mitochondrial energy metabolism: Assessment of hippocampal energy metabolism; (J) Mitochondrial respiratory chain complex detection (Complex-I); (K) Mitochondrial respiratory chain complex detection (Complex-III). Values are presented as mean ± SD. All experiments were performed at least three times. #p < 0.05.

To provide additional evidence of the regulatory effects of DOX-induced heart damage on heart muscle cells, we conducted TOM20 immunofluorescence staining and quantified the transcriptional levels of genes associated with FUNDC1-mediated receptor-dependent mitophagy. The experimental results revealed that DOX-induced myocardial injury significantly diminished TOM20 fluorescence expression and decreased the expression levels of FUNDC1 and ATG5. These findings indicate that DOX-induced myocardial injury adversely affects FUNDC1-mediated mitophagy (Figure 6A-D). Significantly, the application of mitochondrial ATP synthase inhibitors and mitophagy inhibitors resulted in a further decrease in FUNDC1-mediated receptor-dependent mitophagy levels (Figure 6A-D). These findings lead us to hypothesize that the DOX-induced reduction in mitophagy levels may be linked to mitochondrial energy metabolism and the synthesis of new mitochondrial DNA.

### SIRT6-Nrf-2 axis regulates NLRP3-mediated inflammatory injury in doxorubicin-induced myocardial damage

To substantiate the aforementioned mechanisms, we developed a cardiomyocyte model with Nrf-2 gene overexpression to assess energy metabolism and mitochondrial respiratory chain function, specifically focusing on Complex I and Complex III, in the context of DOX-induced injury (Figure 6I-K). The experimental findings demonstrated that DOX-induced damage in cardiomyocytes disrupted intracellular homeostasis, resulting in substantial reductions in mitochondrial basal respiration, maximal respiration, and spare respiratory capacity, accompanied by elevated levels of proton leakage. Further evaluation of mitochondrial respiratory chain function revealed a significant decline in the levels of mitochondrial respiratory complexes, specifically Complex I and Complex III, in cardiomyocytes following the establishment of the model (Figure 6I-K).

To investigate the roles of mitophagy and mitochondrial energy metabolism in DOX-induced myocardial injury, we employed a gene-modified model with overexpression of Nrf-2. Notably, the overexpression of Nrf-2 and SIRT6 (ad-Nrf-2 and ad-SIRT6) effectively reversed the dysfunction in mitochondrial energy metabolism and impairments in the respiratory chain caused by DOX. These results indicate a potential interaction between Nrf-2 and SIRT6 in modulating late-stage mitochondrial energy metabolism dysfunction and mitochondrial biosynthesis impairment in DOX-exposed cardiomyocytes (Figure 6A-K). Furthermore, interventions using mitophagy inhibitors led to significant reductions in mitochondrial energy metabolism, suggesting that mitophagy inhibition plays a crucial role in contributing to mitochondrial energy metabolism dysfunction and respiratory chain impairments (Figure 6A-K). This dysfunction may perpetuate a vicious cycle, serving as a critical precondition for inflammatory damage in cardiomyocytes.

Subsequent research findings validated that DOX-induced myocardial damage significantly boosted TNF-α and IL-18 levels in heart muscle cells, along with activating the NLRP3 inflammasome. These observations imply that, under conditions of impaired mitophagy and mitochondrial energy metabolism, mitochondrial-mediated inflammatory factors are further activated, potentially precipitating programmed cell death in cardiomyocytes (Figure 6A-K). Notably, the overexpression of Nrf-2 and SIRT6 counteracted these effects, attenuating the overexpression of inflammatory damage markers mediated by mitochondrial pathways and inhibiting the excessive activation of the NLRP3 inflammasome (Figure 6A-K). Interestingly, interventions utilizing mitophagy inhibitors and mitochondrial ATP synthase inhibitors intensified inflammatory damage, thereby emphasizing the role of mitochondrial inflammatory damage in exacerbating mitochondrial energy metabolism dysfunction and cardiomyocyte injury (Figure 6A-K). These findings highlight the intricate relationship between SIRT6-Nrf-2-mediated regulation of mitophagy, mitochondrial biosynthesis mechanisms, and the progression of DOX-induced myocardial injury.

### SIRT6 regulates Nrf-2-associated mitophagy to suppress DOX-induced myocardial cell pyroptosis

**Figure 7:**
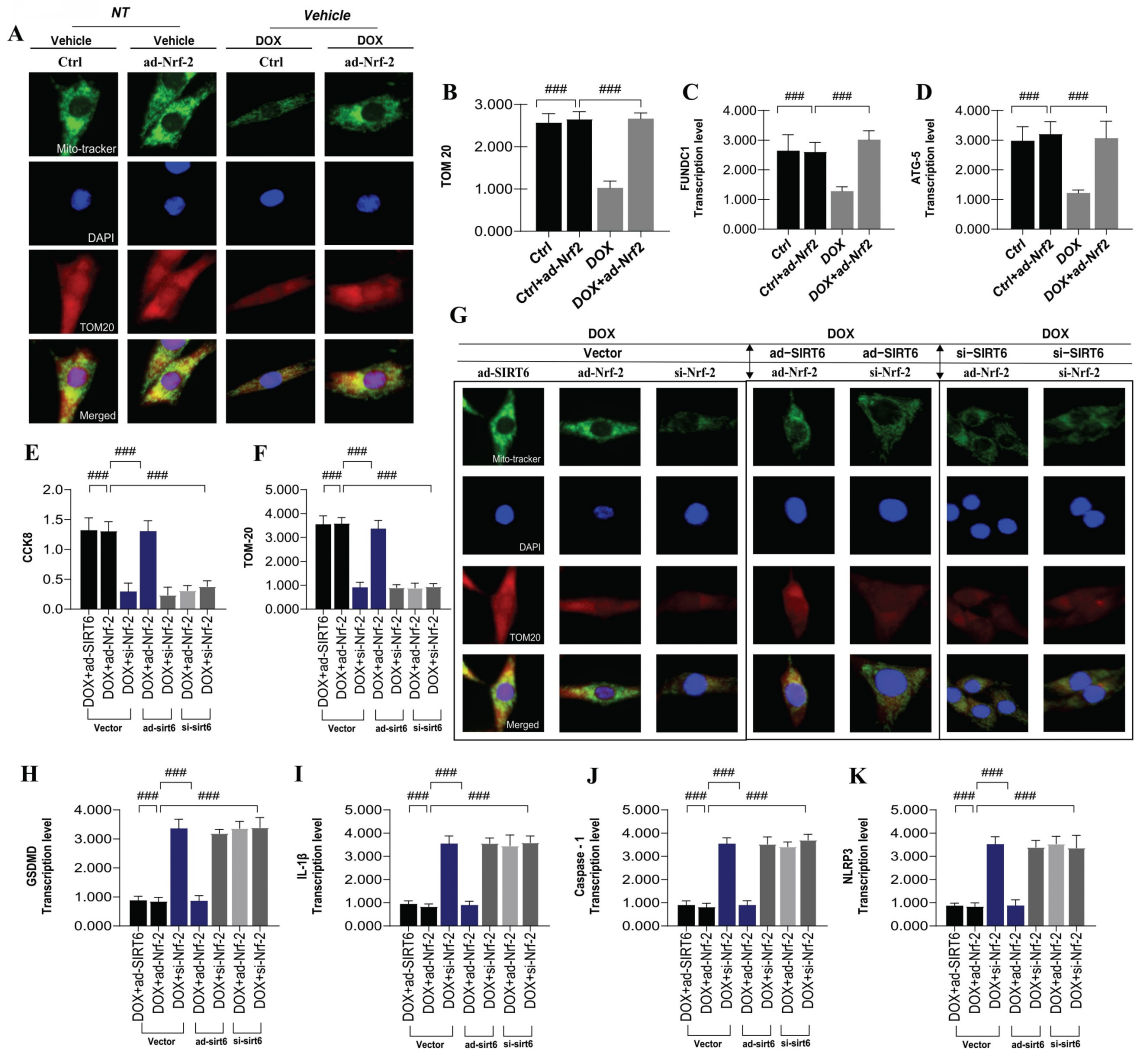
** SIRT6 ameliorates DOX-induced myocardial cell injury by modulating mitochondrial autophagy mediated by Nrf-2/FUNDC1 and blocking pyroptosis.** (A-B) TOM20: Detection of myocardial cell mitophagy levels; (C) FUNDC1: Transcriptional level of FUNDC1; (D) ATG5: Transcriptional level of ATG5; (E) CCK-8: Detection of cell viability using CCK-8 assay; (F-G) TOM20: Detection of myocardial cell mitochondrial autophagy levels; (H-K) Pyroptosis-related gene expression: Transcriptional levels of GSDMD, IL-1β, NLRP3, and Caspase-1; Values are presented as mean ± SD. All experiments were performed at least three times. #p < 0.05.

Building on previous research, we confirmed that mitophagy is primarily mediated by molecules like FUNDC1, which interact with autophagy-related proteins to induce mitophagy^16^, ^28^. This process aids in degrading damaged mitochondria under DOX-induced mitochondrial damage, maintaining cardiomyocyte energy metabolism and preventing programmed cell death. To validate mitophagy's role in DOX-mediated myocardial injury, we used an Nrf-2 overexpression model and assessed TOM20 fluorescence and transcription levels of FUNDC1 and ATG5(Figure 7A-K). Results showed that Nrf-2 overexpression enhanced mitophagy and improved cardiomyocyte viability under DOX-induced injury, indicating a significant correlation between mitochondrial biosynthesis and mitophagy in this context (Figure 7A-K).

Despite the observed transcriptional upregulation of pyroptosis-related genes and increased NLRP3 expression in damaged myocardium, the precise regulatory mechanisms governing pyroptosis remain to be fully elucidated. Pyroptosis, a form of cell death, is dependent on the activation of caspase-1. Under conditions of DOX-induced cardiotoxicity, elevated intracellular ROS production results in the excessive activation of the NLRP3 inflammasome and inflammatory factors, which in turn activate caspase-1- and caspase-8-mediated pyroptotic pathways. To delve deeper into the function of Nrf-2 in controlling pyroptosis during the advanced stages of mitophagy, we analyzed the transcription levels of pyroptosis-related genes, including GSDMD, IL-1β, caspase-1, and NLRP3. The outcomes of our experiments demonstrated that DOX significantly increased the transcription levels of these genes, which was associated with reduced cardiomyocyte viability (Figure 7A-K). However, the overexpression of Nrf-2 and SIRT6 markedly suppressed the activation of the pyroptotic pathway, suggesting that the inhibition and activation of mitophagy are critical determinants of mitochondrial energy metabolism and pyroptosis in DOX-induced pathological states (Figure 7A-K).

Our findings indicate that during the initial phases of cardiomyocyte injury, mitophagy plays a crucial role in diminishing intracellular ROS levels and attenuating NLRP3 inflammasome activation, thereby inhibiting pyroptosis mediated by caspase-1 and caspase-8. Conversely, under conditions of excessive DOX stress, impaired receptor-dependent mitophagy may result in the accumulation of excessive ROS and hyperactivation of the inflammasome, thereby facilitating pyroptosis. Furthermore, our research reveals that SIRT6 exerts a dual function in determining the "survival or death" fate of cardiomyocytes and tumor cells by inducing metabolic reprogramming, characterized by enhanced mitochondrial respiration and suppressed glycolysis. In a model of DOX-induced myocardial injury, overexpression of SIRT6 was found to inhibit glycolysis and promote mitochondrial respiration, thereby reversing the inhibition of ATP production induced by DOX, enhancing the energy supply to cardiomyocytes, and alleviating stress responses. Nonetheless, the regulatory mechanisms that connect mitochondrial biosynthesis to energy metabolism following mitophagy remain to be elucidated. To explore this, we employed genetically modified cardiomyocyte models to examine the combined effects of SIRT6 and Nrf-2 on DOX-induced mitochondrial injury (Figure 7A-K).

Experimental results demonstrated that under ad-SIRT6 conditions, altering Nrf-2 levels (via ad-Nrf-2 or si-Nrf-2) did not impact its regulatory role in mitophagy and pyroptosis (Figure 7A-K). However, under si-SIRT6 conditions, Nrf-2 modulation inhibited its regulatory function in these processes (Figure 7A-K). This suggests that SIRT6 and Nrf-2 interact synergistically, affecting mitochondrial autophagy and biosynthesis networks, which are crucial in mitochondrial energy metabolism dysfunction and pyroptosis during DOX-induced cardiotoxicity. In conclusion, SIRT6 and Nrf-2 play critical roles in managing mitochondrial energy metabolism, autophagy, and pyroptosis in DOX-mediated myocardial injury, highlighting their interaction as vital for reducing DOX cardiotoxicity.

## Discussion

Despite the well-documented cardiotoxicity associated with DOX and related anthracycline anticancer drugs, these agents remain prevalent in the clinical management of various malignancies^31^. Given the extensive population of cancer patients undergoing DOX therapy, even a low incidence of cardiomyopathy translates into a significant number of affected individuals. Doxorubicin, as a prototypical anthracycline antitumor antibiotic, is characterized by cardiotoxicity as a major adverse effect, which severely restricts its clinical utility and adversely affects patients' quality of life. Dysfunction in mitochondrial energy metabolism, driven by morphological and structural damage, constitutes a fundamental pathological mechanism of DOX cardiotoxicity. Considering the high energy requirements of the myocardium, cardiomyocytes depend on a rich supply of mitochondria to fulfill their metabolic demands. Cardiomyocytes and coronary endothelial cells sustain mitochondrial homeostasis and energy metabolism through quality control processes^31^-^33^.

Extensive research has established that DOX cardiotoxicity impairs mitophagy and disrupts energy metabolism in cardiomyocytes^34^, ^35^. Restoration of mitophagy and energy metabolic pathways has been demonstrated to mitigate DOX-induced myocardial damage. Recent investigations have proposed various hypotheses to elucidate the mechanisms underlying DOX-mediated cardiotoxicity, including the accumulation of ROS and dysfunction in mitochondrial biosynthesis within cardiomyocytes^36^-^38^. In this study, we identified SIRT6 as a potential biomarker for DOX-induced mitochondrial dysfunction through an analysis of the GEO datasets GSE106297 and GSE157282. Furthermore, SIRT6 was found to be closely associated with oxidative stress. Our study, employing genetic modifications alongside *in vitro* and *in vivo* experiments, identified oxidative stress and inflammatory damage as pivotal factors contributing to DOX-induced mitochondrial dysfunction in cardiomyocytes. *In vivo* experiments indicated that DOX-induced mitochondrial myocardial injury was correlated with a decreased cardiac ejection fraction, exacerbated oxidative stress, and heightened neutrophil-mediated inflammatory damage. However, treatment with SIRT6 transgenics effectively reversed the oxidative and inflammatory damage induced by DOX. Notably, inhibitors of mitochondrial ATP synthase and mitophagy were ineffective in reversing the reduction in ejection fraction or the oxidative stress and mitochondrial damage caused by DOX.

In addition, *in vivo* studies demonstrated that DOX-induced myocardial injury was characterized by cardiomyocyte hypertrophy, cellular damage, and the upregulation of inflammatory markers, such as MMP9, IL17, and the NLRP3 inflammasome. There was also a marked increase in the transcriptional levels of pyroptosis-related genes, including Caspase-1, Caspase-8, and IL-1β. Treatment with SIRT6 transgenic intervention effectively restored normal cardiac ejection function, alleviated myocardial hypertrophy and inflammation, and inhibited pyroptosis. Supporting *in vitro* studies confirmed that DOX-induced damage to cardiomyocytes was associated with reduced mitophagy, heightened inflammation, disrupted mitochondrial energy metabolism, and the triggering of mitochondrial respiratory chain dysfunction and pyroptotic pathways^39^.

Mitochondrial biosynthesis primarily relies on the coordinated action of transcription factors such as PGC1-α, Tfam, Nrf-1, and Nrf-2 to orchestrate the complex construction and remodeling of DNA. This process enables organisms to adapt to regulatory conditions and further optimize biological structures. Previous studies have effectively utilized mitochondrial biosynthesis methods to enhance the regulation and optimization of phenotypic molecules, regulatory proteins, and components/epigenetic functions, thereby improving overall performance and efficacy^40^. Mitochondrial biosynthesis comprises two main aspects: synthesis within the nuclei of cardiomyocytes and synthesis within mitochondria. Nuclear synthesis in cardiomyocytes primarily involves the production of mitochondrial RNA and proteins, whereas mitochondrial synthesis focuses on the replication of mitochondrial DNA (mtDNA) and protein translation. The mtDNA consists of circular DNA and is typically divided into two strands: the heavy strand and the light strand. Accurate replication of both strands is a critical regulatory mechanism in mitochondrial biosynthesis, ensuring the precise duplication of mtDNA molecules^41^. Transcription factors Nrf-1 and Nrf-2 are essential regulators in this process.

Furthermore, mitochondrial protein translation depends on numerous cytoplasmic enzymes and proteins, while enzymes and proteins localized on the mitochondrial inner membrane serve as the primary sites for the functional activity of mitochondrial proteins. Mitochondrial biosynthesis is controlled by a range of elements such as transcription factors, RNA stability factors, helicases, and proteases. These components work together intricately and in a coordinated way to influence mitochondrial biosynthesis. Additionally, mitochondrial biosynthesis is influenced by the cellular environment, signal transduction pathways, and external factors. Disruptions in these processes can lead to cellular nutrient and energy deficiencies, impairing the normal operation of the mitochondrial respiratory chain. Such disturbances may also result in an abnormal surge of mitochondrial ROS, ultimately triggering the activation of mitochondrial-mediated apoptotic pathways^42^.

Although the Nrf-2 transcriptional regulatory mechanism has been preliminarily validated in the context of mitochondrial biosynthesis, the upstream regulatory mechanisms remain unexplained. Furthermore, the specific regulatory targets involved in mitochondrial homeostasis and inflammatory damage have yet to be elucidated. The interaction between SIRT6 and Nrf-2 has been demonstrated to reverse inflammatory damage, enhance mitochondrial energy metabolism and inhibit apoptosis mediated by the mitochondrial pathway^43^. Subsequent investigations have confirmed that SIRT6, via Nrf-2 downstream protein-mediated mitochondrial biosynthesis, suppresses mitophagy, improves mitochondrial energy metabolism, and prevents DOX-induced cardiomyocyte pyroptosis, thereby enhancing cardiac ejection function and mitigating myocardial damage. Previous studies have indicated that DOX-induced excessive accumulation of ROS and oxidative stress compromise mitochondrial DNA integrity and reduce mitochondrial membrane potential, leading to impaired mitochondrial respiratory chain function and decreased ATP synthesis. Furthermore, DOX-induced dysfunction in cardiomyocyte energy metabolism is linked to reduced expression of electron transport chain proteins and the facilitation of mPTP opening, which directly impairs mitochondrial function. Defective mitochondria, being vital in regulating apoptosis and necrosis, ultimately result in the death of heart muscle cells^44^-^46^. These findings suggest that mitochondria serve as a critical regulatory hub in DOX-induced cardiotoxicity, consistent with our research results.

Through an analysis of publicly available single-cell databases, we have substantiated the regulatory influence of mitochondrial damage pathways in the pathological mechanisms underlying DOX-induced injury. Under conditions of DOX-induced stress, mitochondrial dynamics exhibit a pronounced imbalance, characterized by diminished mitochondrial fusion, enhanced fission, and structural aberrations such as mitochondrial fragmentation, cristae distortion, and reduced cristae density. This disequilibrium is primarily attributed to the overactivation of Drp1, which markedly diminishes the expression of mitochondrial fusion-related proteins. During the later stages of mitochondrial biosynthesis collapse, the balance of mitochondrial dynamics is disrupted due to the activation of oxidative stress and inflammatory mediators. Under these damaged conditions, mitochondrial fission mediator protein Drp1 becomes excessively activated, accompanied by an increase in the phosphorylation level of Fis1 at the T34 site. This imbalance leads to increased mitochondrial fragmentation, insufficient ATP synthesis, or impaired energy supply, which, as indicated by our findings, results in deficient mitophagy^47^-^50^. Under DOX-induced damage, TOM20 expression decreases along with mito-tracker levels, suggesting that mitochondrial autophagy and energy metabolism are severely impaired as mitochondrial structures deteriorate. This process also facilitates the activation of mitochondrial-mediated apoptotic pathways^51^-^54^.

The stress induced by DOX exacerbates mitochondrial fission, resulting in the accumulation of superfluous mitochondrial fragments and misfolded proteins. This dysfunction hampers mitophagy, thereby impeding the clearance of damaged or fragmented mitochondria, and further disrupts mitochondrial biosynthesis pathways, including the synthesis of new mitochondrial DNA. Consequently, this process activates caspase-1/caspase-8-dependent pyroptotic pathways in cardiomyocytes^55^, ^56^. Our results support these observations, providing additional evidence that overactivation of SIRT6 enhances mitophagy, elevates mitochondrial energy metabolism, and suppresses the transcription of caspase-1 and caspase-8, thereby inhibiting pyroptotic pathways^57^-^63^. Importantly, as the principal functional organelle of the heart, mitochondrial energy metabolism is substantially regulated by mitochondrial DNA biogenesis, a crucial process for sustaining mitochondrial self-renewal and functionality^64^-^66^. A prior investigation demonstrated that embryonic stem cells and tumor cells deficient in SIRT6 exhibit enhanced glycolysis and compromised mitochondrial respiratory metabolism, mirroring the mitochondrial energy metabolism phenotype observed in DOX-induced injury in the present study. In our research, we observed that SIRT6 overexpression augments mitochondrial energy metabolism during DOX-induced myocardial injury, restores mitochondrial respiratory chain function. This metabolic transition mitigates DOX-induced glycolysis and reverses DOX-mediated mitochondrial respiratory dysfunction. In the heart, fatty acids constitute the primary energy source, while glucose contributes to 30% or more of the total cardiac energy demand. Significantly, 95% of ATP in cardiomyocytes is produced through mitochondrial respiration, and glycolysis accounts for the last 5%. In pathological states, enhanced glycolysis and reduced lactate removal lead to lactate buildup in the heart muscle, which mediates cardiac metabolic dysfunction and promotes the secretion of inflammatory factors and ROS. Consequently, this exacerbates the progression of oxidative stress and inflammatory damage. Therefore, during the recovery phase from doxorubicin-induced myocardial injury, the shift in metabolism from glycolysis to mitochondrial respiration mediated by SIRT6 appears to better support cardiomyocyte survival.

Previous research on the SIRT family has demonstrated that in a model of heart failure induced by transverse aortic constriction (TAC), the expression levels of SIRT5 are markedly diminished. This reduction is associated with decreased SIRT5-mediated desuccinylation of IDH2, elevated ROS levels in myocardial tissue, and significantly impaired mitochondrial functions, including basal respiration, maximal respiration, and respiratory reserve capacity, ultimately leading to severe cardiac ejection dysfunction^67^. Conversely, treatment with mitochondrial-targeted agents such as quercetin significantly upregulated SIRT5 expression, enhanced mitochondrial energy metabolism, and ameliorated cardiac ejection function and myocardial damage. These protective effects were absent in SIRT5 knockout models, underscoring the role of SIRT5 as a vital protective protein and therapeutic target for myocardial injury mediated by mitochondrial pathways. Furthermore, overexpression of SIRT5 was found to suppress aberrant activation of DNA-PKcs, inhibit the overexpression of Drp1 and Fis1, maintain mitochondrial morphological and structural integrity, and prevent apoptosis mediated by mitochondrial pathways^19^.

These findings are consistent with our results, providing additional confirmation of the protective roles of SIRT6 and SIRT5 in stress-induced myocardial injury. Their common capacity to enhance mitochondrial function and reduce cellular damage highlights their potential as therapeutic targets. In a related study, the absence of Nrf-2 was found to be associated with hypoxia-induced mitochondrial dysfunction and necroptotic cardiomyocyte death. Additionally, there was a significant reduction in Nrf-2-related mitochondrial biosynthesis. Notably, there was a marked increase in PINK-Parkin-mediated receptor-independent mitophagy, indicating that mitophagy dysfunction may occur during the later stages of cellular injury. This phenomenon is accompanied by severe impairments in mitochondrial DNA synthesis and biosynthesis dysfunction, which are critical factors in inducing caspase-dependent necroptosis in cardiomyocytes^27^, ^68^, ^69^. These findings align with our research, wherein we demonstrated through cellular experiments that activation of Nrf-2 reverses DOX-induced mitophagy and mitochondrial biosynthesis dysfunction. Furthermore, Nrf-2 activation was shown to restore mitochondrial respiratory chain function and inhibit apoptosis mediated by the mitochondrial pathway.

Nrf-2 is a key transcription factor that enhancing the cell's ability to withstand oxidative stress^70^, ^71^. In normal physiological conditions, Nrf-2 is held in the cytoplasm through its interaction with Keap1, stopping it from moving to the nucleus. As a result, Nrf-2 cannot attach to specific DNA promoter sequences in the nucleus. As a result, Nrf-2 cannot attach to certain DNA promoter sequences in the nucleus, like AREs, which prevents the transcription of antioxidant enzymes such as catalase, superoxide dismutase, and heme oxygenase-1. The Nrf-2-ARE-mediated antioxidant signaling pathway is therefore regarded as a fundamental component of the cellular antioxidant defense mechanism^72^, ^73^.

Under conditions of stress, such as ischemia, hypoxia, inflammatory injury, or toxic damage, the synthesis of antioxidant enzymes mediated by Nrf-2 is inadequate^74^, ^75^. This inadequacy results in a failure to neutralize excessive ROS, thereby exacerbating mitochondrial oxidative stress and impairing biosynthetic functions. Our research further demonstrates that Nrf-2 is regulated by the upstream protein SIRT6. In the context of DOX-induced cardiomyocyte injury, SIRT6 interacts with Nrf-2, contributing to mitophagy and dysfunction of the mitochondrial respiratory chain. In cases of myocardial injury, abnormal expression of upstream proteins disrupts Nrf-2 transcription, leading to the aberrant activation of the NLRP3 inflammasome, downstream inflammatory injury pathways, and mitochondrial pyroptosis. In conclusion, under the pathological conditions associated with DOX-induced myocardial injury, there is an aberrant activation of the NLRP3 inflammasome, which leads to decreased expression levels of SIRT6 and Nrf-2, as well as impaired FUNDC1-mediated mitophagy. These disruptions result in mitochondrial respiratory chain dysfunction and diminished mitochondrial energy metabolism, thereby exacerbating myocardial injury and cardiac ejection dysfunction. Our research clarifies how SIRT6 and Nrf-2 work together to reduce heart cell damage caused by DOX. This interaction provides mitochondrial protection by boosting mitophagy and energy metabolism, restoring the function of the mitochondrial respiratory chain, and reducing oxidative stress and inflammation, while also preventing mitochondrial damage. The modulation of DOX-induced myocardial injury through SIRT6-mediated restoration of Nrf-2 function underscores the potential of the SIRT6-Nrf-2 axis as a pivotal therapeutic target for mitigating DOX-induced myocardial injury and its associated cardiac dysfunction.

Although we elucidated the roles of SIRT6 and Nrf-2 in mitochondrial pathways of DOX-induced myocardial injury through *in vitro* and *in vivo* experiments, our study has certain limitations. First, we did not employ genetically modified animal models targeting Nrf-2 to explore the regulatory mechanisms of Nrf-2 dysregulation at the organismal level. In future studies, we will utilize cardiac-specific Nrf-2 knockout and overexpression transgenic mice to further clarify the mechanistic role of Nrf-2 in DOX-induced myocardial injury. Second, single-cell sequencing or spatial transcriptomics technologies were not applied to identify cell abundance or abnormal expression regions in the myocardial injury sites or cell populations. Moving forward, we plan to employ these advanced techniques to investigate the spatial transcriptional alterations and differential expression levels of Nrf-2 or SIRT6 in genetically modified models. Lastly, we did not explore potential therapeutic drugs in depth. Given the prior identification of multiple SIRT6-related drug targets, we aim to conduct further *in vitro* and *in vivo* experiments to explore targeted therapeutics associated with SIRT6. This will facilitate the discovery of promising drugs for the treatment of DOX-induced myocardial injury and provide valuable insights for their clinical translation.

## Supplementary Material

Supplementary figure.

## Figures and Tables

**Table 1:** Dataset information

Dataset	Platform	Count	DOX	Control
GSE157282	GPL24676	6	3	3
GSE106297	GPL11154	12	8	4
