# Supplementary material for: SIRT6 mitigates doxorubicin-induced cardiomyopathy via amelioration of mitochondrial dysfunction: A mechanistic study implicating the activation of the Nrf-2/FUNDC1 signaling axis
Source: Int J Med Sci. 2025 Feb 28;22(7):1640–57. doi: 10.7150/ijms.101520 (PMC11905259; doi:10.7150/ijms.101520)
Supplement: Supplementary file 1 — Supplementary figure. [file ijmsv22p1640s1.pdf]

Figure S1:

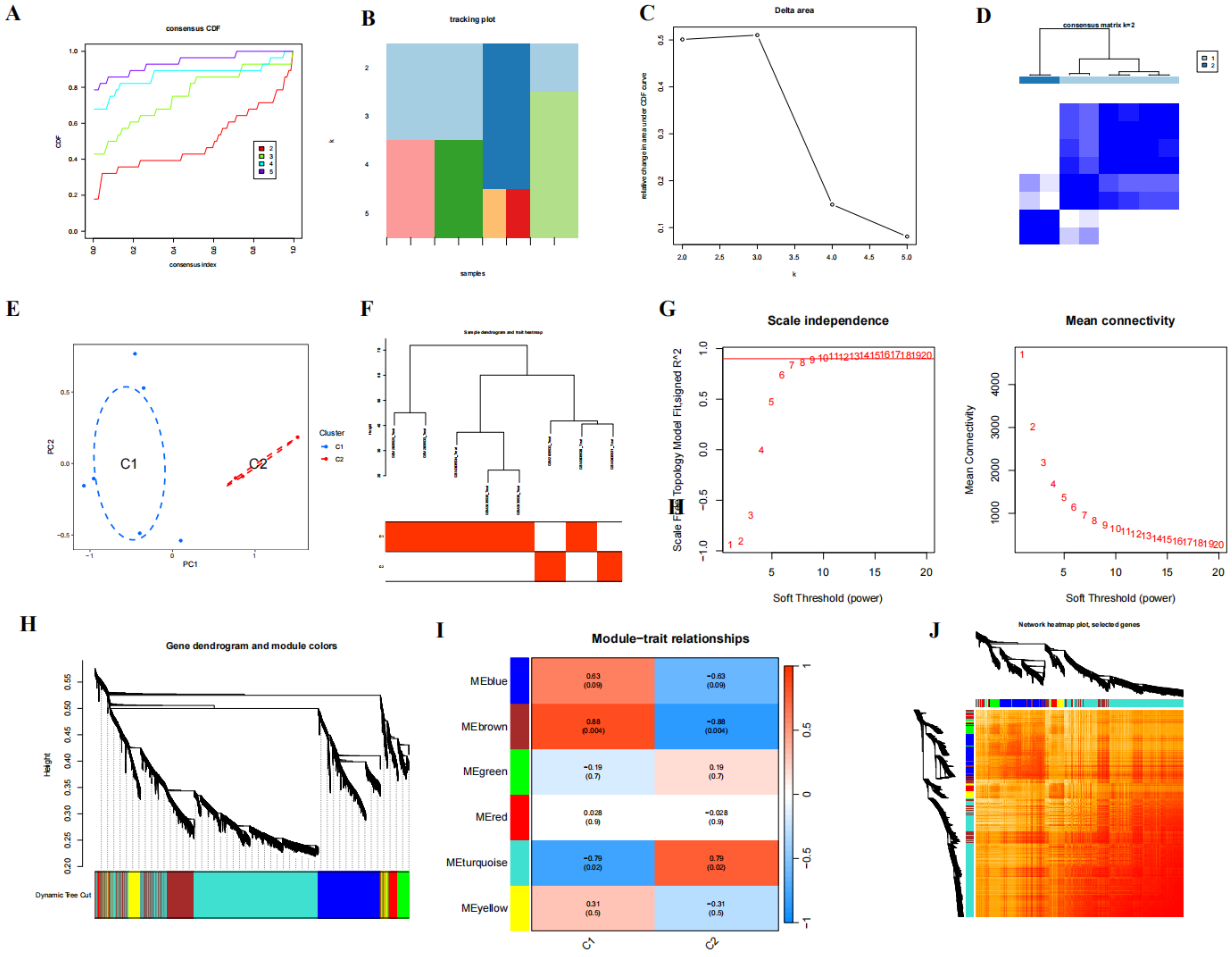

Figure S1: Cluster WGCNA analysis (A-D) Consensus clustering matrix when k = 2. (E) PCA visualized the distribution of the two clusters. (F) Cluster diagram of module eigengenes (G) Set soft threshold power (H) TOM heatmap of 6 modules (I) Heatmap of correlation analysis of module eigengenes with clinical features. Rows and columns represent modules and clinical features, respectively (J) TOM heatmap of 6 modules; When k is set to 2, the highest within-cluster correlation is observed, indicating that MRGs can be used to divide DOX cardiomyopathy samples into two groups (Figure 2A, Figure 2B, Figure 2C, Figure 2D). Figure 2E demonstrates significant differences in principal component analysis (PCA) between these two clusters.
